# New Publications

**Published:** 1851-09

**Authors:** 


*New Publications.*— Several works are on our table, awaiting notice, among which are the able Treatise on Affections of the Urinary System, by Prof. Gross; Dr. Gregory on Eruptive Fevers, edited by Dr. Buckley, and Tilt on Menstruation. We must entreat the indulgence of those who may be interested in this branch of our editorial duties. Were we to act in accordance with the opinion of the late Rev. Sidney Smith, viz., that, to avoid prejudice, it is better to review books *before* reading them, we should have discharged, ere this, our obligations to those who have favored us with new publications. But deeming it necessary to bestow some examination upon new works prior to speaking of their merits, or demerits, we require a larger amount of leisure for editorial notices than suffices to indite them. We hope soon to be able to look beneath the covers of the attractive volumes before us, and to advance beyond the title pages and prefaces.

